# Factors Impacting Academic Productivity and Satisfaction of Surgeon-scientists: A Nationwide Survey

**DOI:** 10.1097/SLA.0000000000006254

**Published:** 2024-02-29

**Authors:** Paula Marincola Smith, Amy Martinez, Rebecca Irlmeier, Carmen C. Solórzano, Deepa Magge, Fei Ye, James R. Goldenring

**Affiliations:** * Section of Surgical Sciences, Vanderbilt University Medical Center, Nashville, TN; † Surgery Division, The University of Texas MD Anderson Cancer Center, Houston, TX; ‡ Office of Research, Vanderbilt University Medical Center, Nashville, TN; § Department of Biostatistics, Vanderbilt University Medical Center, Nashville, TN; ∥ Department of Medicine, Vanderbilt University Medical Center, Nashville, TN; ¶ Nashville VA Medical Center, Nashville, TN

**Keywords:** academic surgery, funding, grant funding, productivity, research, surgeon scientist, surgery

## Abstract

**Objective:**

To identify factors related to research success for academic surgeons.

**Background:**

Many recognize mounting barriers to scientific success for academic surgeons, but little is known about factors that predict success for individual surgeons.

**Methods:**

A phase 1 survey was emailed to department chairpersons at highly funded U.S. departments of surgery. Participating chairpersons distributed a phase 2 survey to their faculty surgeons. Training and faculty-stage exposures and demographic data were collected and compared with participant-reported measures of research productivity. Five primary measures of productivity were assessed, including the number of grants applied for, grants funded, papers published, first/senior author papers published, and satisfaction with research.

**Results:**

Twenty chairpersons and 464 faculty surgeons completed the survey, and 444 faculty responses were included in the final analysis. Having a research-focused degree was significantly associated with more grants applied for [Doctor of Philosophy, incidence rate ratio (IRR) = 6.93; Masters, IRR = 4.34] and funded (Doctor of Philosophy, IRR = 4.74; Masters, IRR = 4.01) compared with surgeons with only clinical degrees (all *P* < 0.01). Having a formal research mentor was significantly associated with more grants applied for (IRR = 1.57, *P* = 0.03) and higher satisfaction in research (IRR = 2.22, *P* < 0.01). Contractually protected research time was significantly associated with more grants applied for (IRR = 3.73), grants funded (IRR = 2.14), papers published (IRR = 2.12), first/senior authors published (IRR = 1.72), and research satisfaction (odds ratio = 2.15; all *P* < 0.01). The primary surgeon-identified barrier to research productivity was lack of protection from clinical burden.

**Conclusions:**

Surgeons pursuing research-focused careers should consider the benefits of attaining a research-focused degree, negotiating for contractually protected research time, and obtaining formal research mentorship.

Historically, surgeons have made impactful contributions to science and medicine outside of the operating room.^1^ Despite this legacy, academic surgeons in the 21st century struggle to generate impactful research while remaining clinically active.^2–12^ Opinion pieces on this topic^12–16^ mostly focus on the increased competitiveness of National Institutes of Health (NIH) research funding juxtaposed with increasing pressure by academic medical centers for surgeons to maintain clinical productivity. Many have cited a mismatch in incentives, with significant incentives to increase patient encounters/billing and minimal incentive to take time away from these efforts for research.^10,14,16–18^ Incentivization programs have been proposed to shift surgeons’ priorities toward academic (rather than clinical) productivity.^19–22^

However, few studies have examined academic surgeon-specific factors that predict research productivity and satisfaction. In this survey project, we examined factors that contribute to or hinder research success for surgeons at top departments of surgery in the United States.

## METHODS

We designed a 2-tiered survey to engage with Department of Surgery chairpersons and faculty at the 30 U.S. departments of surgery with the highest levels of NIH funding according to the 2021 Blue Ridge Medical Research Database (http://www.brimr.org/NIH_Awards/2021/default.htm). Surveys were designed with the assistance of the Vanderbilt Survey Research Shared Resource and approved by the Vanderbilt University Institutional Review Board (May 4, 2022, IRB #220162).

The phase 1 survey (Appendix 1, Supplemental Digital Content 1, http://links.lww.com/SLA/F34) was distributed through email to the Department of Surgery chairpersons at selected institutions and examined chairperson priorities, department-level resource allocation, and incentive structures. Participating chairpersons were asked to forward the phase 2 survey (Appendix 2, Supplemental Digital Content 1, http://links.lww.com/SLA/F34) to faculty surgeons in their departments. All faculty surgeons were invited to participate. The phase 2 survey collected information on surgeon experience, training, interests, and other personal/professional factors. Surveys were anonymous and all questions were optional. Surgeons were incentivized to participate with a $20 e-gift card. Data collection ran from June 2022 to February 2023. Survey responses were manually reviewed for quality control. Survey responses from nonsurgeons [eg, Doctor of Philosophy (PhD)-only research scientists] were excluded.

Faculty research productivity was measured by 5 primary outcomes of interest: (1) number of grants applied for, (2) number of grants funded, (3) number of papers published, (4) number of first/senior author papers published, and (5) satisfaction attaining research goals. In our study, grants included NIH/federal, foundation/nonfederal, and internal/institutional awards. A tabulation of the funded grant types that participants reported can be found in Supplemental Table 1 (Supplemental Digital Content 1, http://links.lww.com/SLA/F34).

Descriptive statistics were used to summarize department and faculty characteristics. Data are reported as median (Q1–Q3) and count (%) for continuous and categorical variables, respectively. Associations between faculty characteristics and outcomes were assessed with zero-inflated negative binomial mixed-effect models for the 4-count outcomes and mixed-effect ordinal models for the satisfaction outcome. All analyses were adjusted for years since training, and the department was treated as a random-effect factor to account for any correlation in respondent outcomes within institutions. We conducted a complete case analysis since we were unable to assume missing at random. *P* values were considered significant at the 2-sided 0.05 level. R version 4.2.2 was used for all statistical analyses.

## RESULTS

Twenty of 30 (66.7%) department chairpersons completed our phase 1 survey. Of these, 13 (65.0%) distributed the phase 2 survey to their faculty with 464 total phase 2 survey responses received. After quality control, 20 phase 2 surveys were excluded and 444 were included in our analysis.

### Chairperson Responses

Nineteen (95.0%) chairpersons reported basic science as either a high or essential priority for their department, with 18 (90.0%) reporting the same for clinical outcomes, 17 (85.0%) for clinical trials, 15 (75.0%) for diversity/equity and inclusion, and 15 (75.0%) for health equity research (Supplemental Digital Content Fig. 1, http://links.lww.com/SLA/F34).

Nineteen (95.0%) chairpersons reported offering their faculty grant management services, 19 (95.0%) direct early career funding, 18 (90.0%) grant writing workshops, 18 (90.0%) biostatistics support, 17 (85.0%) formal research mentorship, and 17 (85.0%) clinical trials support services.

Twelve (60.0%) chairpersons reported directly incentivizing grants awarded, whereas fewer incentivize manuscript publications (5, 25.0%) or presenting at national meetings (3, 15.0%). Ten (50.0%) chairpersons reported having benchmarks for grant funding, whereas fewer have benchmarks for manuscripts published (7, 35.0%) or national presentations (4, 20.0%) (Supplemental Digital Content Table 2, http://links.lww.com/SLA/F34) Of note, benchmarks differed from chairperson to chairperson. The range of benchmarks for grants, publications, and presentations outlined by different chairperson respondents can be reviewed in the complete, publicly available, data set.

### Faculty Respondent Characteristics

Faculty respondents represented surgeons from 13 institutions with respondents per institution ranging from 12 to 86 (median 24, 8–44), as well as 25 respondents who chose not to disclose their institution. Characteristics of faculty respondents are shown in Supplemental Table 3 (Supplemental Digital Content 1, http://links.lww.com/SLA/F34). Most respondents were males (281, 63.3%), White (325, 73.2%), non-Hispanic/Latino (394, 88.7%), and had children/dependents (353, 79.5%). Respondents represented 24 surgical specialties and subspecialties (Supplemental Digital Content Fig. 2, http://links.lww.com/SLA/F34). In addition to a clinical degree, 170 (38.3%) respondents reported having a research-focused degree, including 77 (17.3%) Master of Science, 44 (9.9%) Master of Public Health, 38 (8.6%) PhD, 8 (1.8%) Master of Science in Clinical Investiagition, and 3 (0.7%) Master of Arts.

Respondents were a median of 10.0 years (4.0–20.0) out from completing training and included 8 (1.8%) instructors, 168 (37.8%) assistant professors, 117 (26.4%) associate professors, and 142 (32.0%) professors.

### Faculty Respondent Research Experience and Training

The majority (260, 58.6%) of faculty respondents completed dedicated research time (≥1 year) during their residency (Table 1).

**TABLE 1:** Summary of Research Training and Experience by Faculty Respondents

	No. of respondents (N = 444)	
	Count (n)	(%)
Completed dedicated research time during residency		
No	139	31.3
Yes	298	67.1
Missing	7	1.6
Dedicated research time during residency (yr)		
0	139	31.3
<1	38	8.6
1–<2	87	19.6
2–<3	133	30.0
3–<4	37	8.3
4+	3	0.7
Missing	7	1.6
Completed surgical fellowship training		
No	49	11.0
Yes	384	86.5
Missing	11	2.5
Completed dedicated research time during fellowship (mo)		
0	276	62.2
1–<3	62	14.0
3–<6	15	3.4
6–<9	6	1.4
9–<12	6	1.4
12+	17	3.8
Missing	62	14.0
Research Productivity During Training		n
Grants applied for during residency, median (Q1–Q3)	1.0 (0.0–2.0)	292
Grants successfully funded during residency; median (Q1–Q3)	1.0 (0.0–2.0)	193
Papers published during residency; median (Q1–Q3)	10.0 (4.0–20.0)	193
First/senior author papers during residency; median (Q1–Q3)	5.0 (3.0–10.0)	185
Grants applied for during fellowship; median (Q1–Q3)	0.0 (0.0–1.0)	101
Grants successfully funded during fellowship; median (Q1–Q3)	1.0 (0.0–2.0)	39
Papers published during fellowship; median(Q1–Q3)	4.0 (2.0–8.0)	106
First/senior author papers published during fellowship; median (Q1–Q3)	3.0 (1.0–5.5)	95

The most common types of research respondents engaged in during residency included basic science (194, 43.7%), clinical outcomes (131, 29.5%), translational science (55, 12.4%), quality improvement (30, 6.8%), and clinical trials (25, 5.6%).

Of the 384 faculty respondents (86.5%) who completed clinical fellowship training, 106 (23.9%) reported completing dedicated research time (≥1 month) during the fellowship. The most common types of research engagement during fellowship included clinical outcomes (75, 16.9%), basic science (41, 9.2%), and clinical trials (17, 3.8%).

**FIGURE 1:**
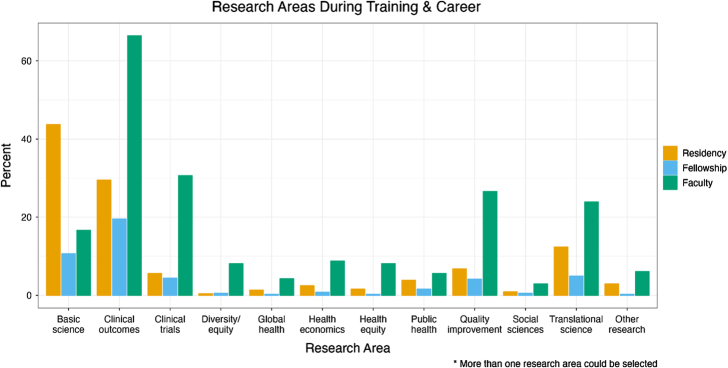
Research areas during training and career. Bar graph demonstrating the percentage of respondents who indicated they engaged in each area of research at various stages of their career (residency, fellowship, and faculty). Respondents could select more than one area of research at each stage of their career.

Faculty respondents reported currently being engaged most in clinical outcomes (295, 66.4%), clinical trials (136, 30.6%), quality improvement (118, 26.6%), translational (106, 23.9%), and basic science (74, 16.7%) research (Fig. 1).

### Research Productivity During Training

Faculty respondents reported a median of 1.0 (0.0–2.0) grants applied for, 1.0 (0.0–2.0) grants funded, 10.0 (4.0–20.0) papers published, and 5.0 (3.0–10.0) first/senior author papers published during residency.

Faculty respondents reported a median of 0.0 (0.0–1.0) grants applied for, 1.0 (0.0–2.0) grants funded, 4.0 (2.0-8.0) papers published, and 3.0 (1.0–5.5) first/senior author papers published during the fellowship.

### Research Productivity of Faculty Respondents

Faculty respondents reported having applied for a median of 3.0 (0.0–10.0) grants, receiving 3.0 (1.0–5.0) grants, publishing 20.0 (2.0–80.0) papers, and publishing 18.0 (5.0–50.0) first/senior author papers since completing training.

When asked about their satisfaction in attaining research goals, 60 (13.5%) reported being “very satisfied,” 132 (29.7%) “satisfied,” 116 (26.1%) “neither satisfied nor unsatisfied,” 65 (14.6%) “unsatisfied,” and 22 (5.0%) “very unsatisfied.”

The number of grants applied for/funded, the number of papers and first/senior author papers published, and satisfaction with research, all increased as respondents progressed through the ranks from assistant to full professor. While assistant professors reported a median of 1.0 (1.0–3.5) grants funded and 3.0 (0.8–9.2) first/senior author papers published, associate professors reported 2.0 (1.0–5.0) grants funded and 20.0 (6.0–30.0) first/senior author papers published, and professors reported 4.0 (2.0–10.0) grants funded and 50.0 (25.0–80.0) first/senior author papers published (Table 2).

**TABLE 2:** Summary of Self-reported Research Productivity by Faculty Respondents by Rank

	Assistant professor (N = 168)	Associate professor (N = 117)	Professor (N = 142)	Overall (N = 444)
Grants applied for as faculty				
Mean (SD)	4.3 (8.4)	8.5 (13.0)	14.7 (19.9)	8.7 (14.8)
Median (Q1, Q3)	1.0 (0.0, 4.0)	4.0 (1.0, 10.0)	8.0 (2.0, 20.0)	3.0 (0.0, 10.0)
Missing (%)	17 (10.1)	10 (8.5)	14 (9.9)	42 (9.5)
Grants successfully funded as faculty				
Mean (SD)	2.4 (2.9)	3.5 (4.2)	7.8 (10.5)	5.0 (7.6)
Median (Q1, Q3)	1.0 (1.0, 3.5)	2.0 (1.0, 5.0)	4.0 (2.0, 10.0)	3.0 (1.0, 5.0)
Missing (%)	89 (53.0)	36 (30.8)	31 (21.8)	166 (37.4)
Papers published as faculty				
Mean (SD)	10.7 (21.7)	36.9 (36.4)	122.4 (114.4)	55.6 (87.8)
Median (Q1, Q3)	3.0 (0.0, 10.0)	30.0 (10.0, 54.5)	100.0 (43.8, 150.0)	20.0 (2.0, 80.0)
Missing (%)	19 (11.3)	11 (9.4)	14 (9.9)	46 (10.4)
First/senior author papers published as faculty				
Mean (SD)	7.8 (12.6)	22.5 (23.3)	66.5 (63.8)	35.4 (49.8)
Median (Q1, Q3)	3.0 (0.8, 9.2)	20.0 (6.0, 30.0)	50.0 (24.0, 80.0)	18.0 (5.0, 50.0)
Missing (%)	92 (54.8)	47 (40.2)	49 (34.5)	201 (45.3)
Satisfaction attaining research goals; n (%)				
Very satisfied	13 (7.7)	15 (12.8)	30 (21.1)	60 (13.5)
Satisfied	39 (23.2)	32 (27.4)	58 (40.8)	60 (29.7)
Neither satisfied nor unsatisfied	49 (29.2)	33 (28.2)	28 (19.7)	132 (29.7)
Unsatisfied	32 (19.0)	19 (16.2)	11 (7.7)	116 (26.1)
Very unsatisfied	11 (6.5)	5 (4.3)	4 (2.8)	65 (14.6)
Missing (%)	24 (4.3)	13 (11.1)	11 (7.7)	22 (5.0)

**FIGURE 2:**
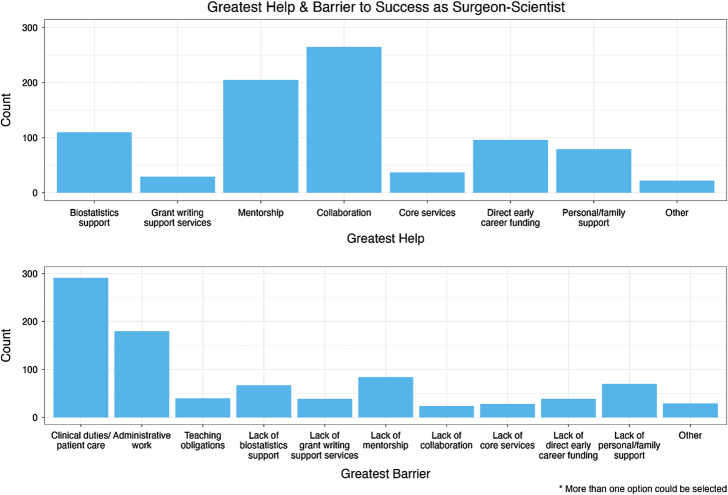
Greatest help and barrier to success as a surgeon-scientist. Number of respondents indicated the various factors that have been the greatest help and greatest barrier to their academic success as a surgeon-scientist. Respondents could select more than one factor as a great help or barrier to their career.

Faculty respondents indicated that collaboration (265, 59.7%), mentorship (205, 46.2%), and biostatistics support (110, 24.8%) had the greatest positive impact on their success as a surgeon-scientist, whereas clinical duties/patient care (291, 65.5%) and administrative work (180, 40.5%) were barriers to academic success (Fig. 2). Faculty were, in addition, asked to provide free text comments regarding factors that contribute to or hinder academic productivity. Of the 123 (27.7%) respondents who provided a free text response, the most common themes were lack of “truly protected” research time and the impact of the clinical/paraclinical burden (39, 31.7%), the perception that the institution does not value research as much as clinical productivity (38, 30.9%), lack of access to support services (31, 25.2%), and lack of mentorship/collaboration (29, 23.6%; Table 3).

**TABLE 3:** Themes from Free-text Faculty Responses Regarding Factors That Contribute to or Hinder Academic Productivity

Theme	n (%)
“Truly protected” time/clinical + paraclinical burden	39 (31.7)
Institution that values research as much as clinical productivity	38 (30.9)
Access to support resources (grant writing, biostats, core services, research team)	31 (25.2)
Mentorship/collaboration with senior scientists	29 (23.6)
Direct institutional research support	19 (15.5)
“Self-motivation”, willingness to sacrifice personal time	17 (13.8)
“Start early”	8 (6.5)
“My kind of research” not valued	7 (5.7)
Bias, DEI, or sex issues	4 (3.3)
Regulatory/administrative burden	4 (3.3)
Grant funding is too competitive	3 (2.4)
Other/unable to classify	22 (17.9)

DEI indicates diversity, equity, and inclusion.

### Primary Analysis

We examined how training factors, including grants applied for, grants funded, papers published, and first/senior author papers published during training, correlated with our primary outcomes of interest. After controlling for years since completing training, only first/senior author papers published during training were significantly correlated with our primary outcomes of interest, and the incidence rate ratios (IRRs) were low: 1.06 (*P* = 0.015) for grants applied, 1.06 (*P* = 0.013) for grants funded, 1.07 (*P* = 0.014) for papers published, and 1.06 (*P* = 0.015) for first/senior author papers published as faculty. First/senior author papers published during training were not significantly associated with satisfaction in research [odds ratio (OR) = 1.02, *P* = 0.559].

We next examined whether having a research-focused degree was significantly associated with our primary outcomes of interest. After controlling for years since completing training, having a research-focused degree was significantly correlated with a higher number of grants applied for (IRR = 4.98, *P* < 0.001) and grants funded (IRR = 4.15, *P* = 0.002). When examining faculty members with Masters versus PhD-level research degrees, we observed a dose-response relationship with PhD-carrying surgeons demonstrating higher IRRs than Masters-carrying surgeons in applying for and obtaining grant funding. There was a similar trend towards higher levels of publications, first/senior author publications, and satisfaction in research with surgeons having PhDs demonstrating higher IRRs/ORs than those with Master’s degrees; however, these relationships were not statistically significant.

Although mentorship was a leading self-reported factor in faculty research success, the impact of having a formal research mentor on our primary outcomes varied. Having a formal research mentor was associated with more grants applied for (IRR = 1.57, *P* = 0.031) and higher levels of satisfaction in research (OR = 2.22, *P* = 0.004), but was not significantly associated with the number of grants awarded, papers published, or first/senior author papers published.

Two hundred two (45.5%) respondents reported having protected time for research (>0%) in their employment contracts. Those with protected time were more likely to apply for grants (IRR = 3.73), have grants funded (IRR = 2.14), publish papers (IRR = 2.12), publish first/senior author papers (IRR = 1.72), and have higher levels of satisfaction in research (OR = 2.15; all *P* ≤ 0.001) than those without protected time. In addition, for every 10% increase in contractually protected research time, there was an incremental increase in our primary outcomes of interest: grants applied for (IRR = 1.34), grants funded (IRR = 1.22), papers published (IRR = 1.22), first/senior author papers published (IRR = 1.10), and satisfaction in research (OR = 1.34; all *P* < 0.001; Supplemental Digital Content Fig. 3, http://links.lww.com/SLA/F34).

Respondents who reported being “satisfied”/“extremely satisfied” in attaining their research goals were significantly more likely to apply for grants (IRR = 2.40, *P* < 0.001), obtain grant funding (IRR = 2.00, *P* < 0.001), publish papers (IRR = 1.95, *P* < 0.001), and publish first/senior author papers (IRR = 1.58, *P* = 0.022) compared with those who were “unsatisfied”/“extremely unsatisfied” (Table 4).

**TABLE 4:** Primary Analyses Examining Relationship Between Individual Factors and Academic Productivity

	Grants applied for as faculty, IRR (CI), *P*	Grants funded as faculty, IRR (CI), *P*	Papers published as faculty, IRR (CI), *P*	First/senior author papers published as faculty, IRR (CI), *P*	Satisfaction in research as faculty, OR (CI), *P*
Funding success as a trainee					
Grants applied for during training	1.04 (0.94–1.14), 0.47	0.99 (0.92–1.07), 0.85	1.03 (0.95–1.12), 0.48	0.96 (0.89–1.03), 0.26	0.95 (0.82–1.10), 0.51
Grants successfully funded during training	0.93 (0.74–1.17), 0.53	1.05 (0.87–1.28), 0.61	0.86 (0.71–1.05), 0.14	1.04 (0.86–1.26), 0.70	1.14 (0.85–1.54), 0.38
Papers published during training	0.98 (0.95–1.00), 0.09	0.98 (0.95–1.00), 0.07	0.98 (0.95–1.01), 0.26	0.98 (0.95–1.01), 0.26	1.01 (0.97–1.06), 0.56
First/senior author papers published during training	**1.06** (**1.01**–**1.12), 0.02**	**1.06** (**1.01**–**1.10), 0.01**	**1.07** (**1.01**–**1.12), 0.01**	**1.06** (**1.01**–**1.12), 0.02**	1.02 (0.95–1.10), 0.56
Research-focused degree (relative to MD-only)					
MD + PhD	**6.93** (**3.04**–**15.78), <0.01**	**4.74** (**1.78**–**12.60), <0.01**	1.99 (0.82–4.84), 0.13	1.57 (0.56–4.37), 0.39	2.43 (0.75–7.89), 0.14
MD + Masters	**4.34** (**2.06**–**9.17), <0.01**	**4.01** (**1.61**–**10.01), <0.01**	1.72 (0.77–3.81), 0.19	1.49 (0.59–3.79), 0.40	1.48 (0.53–4.10), 0.46
Formal research mentor (relative to no formal research mentor as faculty)					
Having a formal research mentor as a faculty	**1.57** (**1.04**–**2.35), 0.03**	1.34 (0.94–1.91), 0.10	0.99 (0.68–1.43), 0.96	0.96 (0.65–1.42), 0.86	**2.22 (1.30**–**3.79), <0.01**
Contractually protected research time (relative to no contractually protected research time)					
Contractually protected research time, yes	**3.73** (**2.65**–**5.27), <0.01**	**2.14** (**1.59**–**2.87), <0.01**	**2.12** (**1.60**–**2.80), <0.01**	**1.72** (**1.24**–**2.38), <0.01**	**2.15 (1.42**–**3.28), <0.01**
Satisfaction attaining research goals (relative to unsatisfied/extremely unsatisfied)					
Neither satisfied nor unsatisfied	1.09 (0.70–1.70), 0.71	1.11 (0.73–1.70), 0.61	0.94 (0.63–1.40), 0.75	0.90 (0.58–1.41), 0.65	NA
Satisfied/extremely satisfied	**2.40** (**1.61**–**3.58), <0.01**	**2.00** (**1.39**–**2.87), <0.01**	**1.95** (**1.37**–**2.79), <0.01**	**1.58** (**1.07**–**2.33), 0.02**	NA

Statistical significance (*P* < 0.05) values are in bold.

CI indicates confidence interval; IRR, incidence rate ratio; MD, Doctor of Medicine; NA, not applicable; OR, odds ratio; PhD, Doctor of Philosophy.

### Secondary Analyses

Secondary analyses were also performed on a variety of demographic factors. The focus of secondary analyses was estimation rather than testing. Secondary analyses of the impact of demographic factors, training factors, and faculty-specific factors are summarized in Supplemental Table 4 (Supplemental Digital Content 1, http://links.lww.com/SLA/F34).

## DISCUSSION

For a multitude of reasons, including ample access to patient tissues, as well as intimate familiarity with disease pathology and therapeutic challenges, surgeons are uniquely positioned to perform impactful research.^1^ However, many surgeons in the 21st century have difficulty maintaining research productivity.^2–11^ This survey was designed to improve our understanding of factors that contribute to or hinder success in research among academic surgeons.

In our survey, obtaining a research-focused degree was significantly associated with a higher likelihood of applying for and receiving extramural research funding, with PhD-carrying surgeons seeing more benefit than those with Masters-level degrees. We, in addition, observed that the association with academic productivity/satisfaction as a faculty member was much weaker for completing dedicated research time during training than for having a research-focused degree, suggesting that completing a research-focused degree itself may position surgeons for academic success in a way that non-degree–focused research time does not. The reason for this may be multifactorial. First, it is possible that surgeon-scientists most dedicated to research will prioritize time, effort, and resources to obtain a degree; thus, having a research-focused degree may simply be a surrogate marker of interest and commitment. Second, many research-focused degrees require rigorous coursework on specific content areas not universally emphasized in medical schools. Critical review of primary literature, grant writing, biostatistics, and other research skills are fundamentally useful when running a research program, and attaining formal training in these areas likely offers a competitive advantage compared with those who may be self-taught in these areas. Those with research-focused degrees may also have access to additional faculty mentors and research networks. Regardless of the reason, our results suggest that surgeons with research-focused degrees may be better positioned to have productive academic careers. Surgeons and surgical trainees interested in a research career may benefit from pursuing a research-focused degree and, conversely, supporting faculty surgeons with research-focused degrees may be in the best interest of academic surgical departments.

We did observe a progression away from basic/translational sciences towards clinical outcomes/clinical trial research as surgeons move through their careers. While 44% of respondents performed basic science research during residency, only 17% of faculty members currently engage in basic science research. Concurrently, involvement in clinical trials and clinical outcomes research increased over time, with 6% versus 31% being involved with clinical trials and 30% versus 66% being involved with clinical outcomes research during residency and as faculty, respectively. Our survey did not directly inquire about what factors may contribute to this shift in research focus. One possibility is that basic science is more accessible at the residency level as residents are often not faced with having to attain independent research funding or manage their own laboratories. Contrast this to the experience of faculty surgeons, who are often faced with high barriers, including setting up, maintaining, and continually funding a functional laboratory. These barriers may be enough to discourage even those with an interest in the basic sciences from participating. If this is the case, creating programs that focus on decreasing barriers to basic science for faculty surgeons such as those that facilitate collaborations with PhD scientists or those that help with start-up funding may be beneficial.

When given a list of options, 66% of faculty respondents indicated that patient care/clinical duties were major barriers to their success as a surgeon-scientist. Similarly, when asked an open-ended question about the biggest barriers to academic productivity, 32% of respondents independently described something related to lack of “truly protected” research time and/or the clinical/paraclinical burden on academic activities. Interestingly, only 45.5% of respondents reported having protected research time outlined in their employment contracts. Those who had any (>0%) protected research time had significantly higher levels of research productivity and satisfaction in research, and increases in all primary measures of academic productivity were observed with incremental increases in contractually protected research time. This suggests that most faculty surgeons who are interested in research are pursuing research without any contractually protected time, which may be a major reason why so many struggle to balance research and clinical obligations. Surgeons who can negotiate faculty contracts may be well-served to advocate for contractually protected research time if research is a priority. Conversely, surgical department chairpersons and deans interested in promoting surgical research should strategically budget and plan with contractual research time for surgical faculty in mind. Of note, the NIH has issued guidance in support of the idea that protected time is critical for the success of the surgeon-scientist, as evidenced by their mandate for ≥50% protected time for surgeon-scientists to be eligible for K awards.^23^

Independent of the type of research being performed, it is clear that real protected time is critical for success, not just initially, but in the long term.^12^ Central to this concept must be a set of realistic expectations between departments and their surgeon-scientist faculty on the levels of clinical work that is required. These expectations should work both ways. Faculty who are seeking long-term 50% protection must recognize that their salary reimbursement will not be as high as their full-time clinical compatriots. In addition, their 50% protection comes with an assumption that these investigators will be successful in obtaining external funding. Similarly, departments should not lure junior faculty in with a limited 50% protection and inflated salary only to require far greater clinical duties after an initial 3 to 4 years to cover the extra salary costs. The departments must value success in research equally with clinical care revenues for a modern academic surgery department to be successful in supporting the endeavors of surgeon-scientists. Conversely, aspiring academic surgeons should consider which element(s) of their career brings them the most satisfaction and the trade-offs inherent in these respective tasks balanced with the need to generate personal income in the context of student loans, etc. The overall meaning/purpose in work^24^ that an individual surgeon derives from the freedom to perform research in a protected space (at a researcher’s salary) versus working (and being reimbursed) as a surgeon is different for everyone, and these factors should be carefully considered when considering a job in academic surgery. Importantly, if all parties, including department leadership, as well as both research and non-research faculty surgeons, view research as fundamental to the mission of the institution and department, then everyone can work together to support a surgeon-scientist who wants to spend time performing research, acknowledging that research is both fundamentally important and costly. Such an arrangement will likely require some degree of salary support for the surgeon-scientist through work relative value unit generation by the full-time clinical faculty. Without these shared values, however, resentment can develop as a result of mismatched expectations with new surgeon-scientist hires expecting equivalent salary and total compensation without contributing to the department’s bottom line similar to their clinical counterparts.

Our findings on the impact of mentorship are worthy of further investigation. Faculty respondents identified mentorship as a leading factor that supports their research careers, and multiple free-text comments indicated that formal mentorship programs are crucial for surgeon-scientist development and advancement. In our study, having a formal mentor was not associated with a significant increase in productivity (grants awarded, publications), yet was associated with higher satisfaction in research. This indicates that respondents may value things other than grants awarded and publications, which could include patient impact, training, mentoring, or work-life harmony. It is important to note that scientists from gender and racial minorities are less likely to receive impactful mentorship^25,26^ and more likely to have negative mentoring experiences.^27^ If mentoring is indeed a primary driver of research success and satisfaction, institutions will benefit from providing equitable access to high-quality mentorship. While the survey did not address patterns of departmental support, patterns of effective mentoring scenarios must be considered. It seems logical to suggest that embedding new surgeon-scientists within the laboratory of a successful mentor likely presents the most efficient approach to early faculty success. We have found at our institution that such embedding of junior faculty for 2 to 3 years optimizes the transitions to success. Having collaborations built into one’s research effort can be beneficial when applying for federal grants that require demonstrated experience and expertise in research techniques, potentially improving chances of funding success for junior faculty. In addition, universities can and should utilize their entire faculties as mentors rather than relying solely on existing laboratories within departments of surgery. This model of embedding new hires into existing laboratories gives the department an extended time to establish and equip laboratory space for junior faculty, while simultaneously allowing departments more time to evaluate potential for career development pathways for new faculty. Importantly, there is likely to be considerable interinstitutional variability in experience with fostering these sorts of collaborations, and faculty applying for surgeon-scientist positions should look for examples of successful collaboration and take this into account as they consider their job prospects.

Another important observation is that we see a relatively high percentage of respondents expressing dissatisfaction in attaining their research goals, particularly for more junior respondents. While 25% of assistant professors reported being either “unsatisfied” or “very unsatisfied” in attaining their research goals, 20% of associate professors and 10% of professors reported the same. This suggests that research satisfaction may increase over the arc of a surgeon-scientist career, or that the beginning of one’s academic career is a particularly stressful or difficult time. Reasons for this could include the challenge and stress of establishing a new operative practice, referral base, and clinical reputation while simultaneously working to establish research collaborations, funding, and setting up a functioning research program. These findings are important and suggest that resources and support focused on junior faculty could help to alleviate some of the stress inherent in being a junior faculty member and potentially improve retention and interest among surgeon-scientists.

Interestingly, 31% of faculty survey respondents reported completing <1 year of dedicated research time during residency. Of note, nearly half of the respondents who did <1 year of dedicated research time during residency are from subspecialties that do not classically do extended periods of research during residency, including Otolaryngology, Oral/Maxillofacial Surgery, Orthopedics, Urology, etc, as opposed to general surgery-based specialties, where extended research time is more common during residency. However, surgeons who completed <1 year of dedicated research time during residency overall appear to be more likely hired into a clinician-educator track versus a research track, appear less likely to engage in basic/translational science research, and appear to have begun identifying as a surgeon-scientist later in their careers compared with respondents who completed ≥1 year of dedicated research time during residency. Based on currently published data, it is unclear what percentage of practicing academic surgeons across all subspecialties in the United States have completed dedicated research time ≥1 year during residency training; however, several studies suggest that those who do complete dedicated research time are more likely to complete fellowship training, choose academic careers, and receive grant funding.^28–32^ At our institution, a Physician Scientist Institutional Award funded by the Burroughs Wellcome Fund allows us to provide surgery residents with funding for their basic/translational science research in the form of technician salary and supplies in their mentor’s laboratory for 2 years after returning to clinical training following dedicated research time. This funding has allowed residents to maintain their research productivity for an extended period of time and to learn early how to navigate the challenges of balancing clinical and research obligations. We believe that similar experiences may lead to greater long-term success in pursuing surgeon-scientist careers in basic and translational research, although long-term data are lacking. Importantly, leaders in academic surgery including members of the American Surgical Association and the American College of Surgeons are coming together to form a second Blue Ribbon Committee on education and research in general surgery training.^33,34^ These sorts of collaborations at the national level which foster thoughtful discussion around many of the issues discussed in this paper are likely to benefit future generations of aspiring surgeon-scientists.

This survey project does have limitations that limit the scope of our conclusions. First and foremost, although this remains one of the largest surveys of academic surgeons to date, we had limited power to perform certain valuable subanalyses. For instance, only 11 (2.5%) of respondents reported being Black/African American, 17 (3.8%) reported being Hispanic/Latino, and 2 (0.5%) reported being gender non-binary. This is, unfortunately, likely reflective of the general population of academic surgeons in the United States where minority groups are underrepresented.^35^ Given the small numbers of respondents in these subgroups, it was impossible to perform statistically sound analyses related to these groups, which may be important to understand factors that disproportionately impact underrepresented minority surgeon-scientists. In addition, it should be highlighted that our outcome metrics, including the number of grants applied for/funded and the number of papers published, are imperfect measures of academic success and productivity. Different fields of research enable publications at different rates, and some academic surgeons prioritize other academic efforts (including education or administration) over research. The fifth outcome of interest, which was satisfaction in one’s own research career, was added in part to attempt to capture this latter point. Interestingly, we found that satisfaction in research did significantly correlate with our other 4 outcomes of interest, suggesting that many faculty surgeon respondents measure their own success at least in part by the same variables we included for our analysis. In addition, our outcome metrics do not directly measure scientific impact. Follow-up studies could focus on author H-indices and/or quantity of grant support ($) rather than simply the number of publications/number of grants to shed more light on scientific impact as a measure of academic productivity. Lastly, our survey included only academic surgeons at 20 highly funded academic departments of surgery in the country, and thus our findings may not be generalizable to the surgeon-scientist population at large.

## CONCLUSIONS

Our survey highlighted a few important points. First, clinical/paraclinical obligations and the associated difficulty in finding time for research are major barriers to academic success for many faculty surgeons. Those with contractually protected time for research appear to struggle less with this balance and demonstrate higher rates of grant funding/publication, as well as satisfaction with research. Second, those with research-focused degrees appear to attain higher levels of extramural grant funding compared with their colleagues without research-focused degrees. Lastly, faculty satisfaction in research correlated significantly with mentorship. Together, these findings suggest that considering a research-focused degree and negotiating protected research time with access to structured mentorship may facilitate a productive and satisfying research career for aspiring surgeon-scientists.

## Supplementary Material
