# Supplementary material for: Factors Impacting Academic Productivity and Satisfaction of Surgeon-scientists: A Nationwide Survey
Source: Ann Surg. 2024 Feb 29;281(3):445–53. doi: 10.1097/SLA.0000000000006254 (PMC11809735; doi:10.1097/SLA.0000000000006254)
Supplement: Supplementary file 1 [file sla-281-445-s001.pdf]

Supplemental Content Legend

Item	Caption
**Appendix 1**	Chairperson Survey
**Appendix 2**	Faculty Survey
**Supplemental Table 1**	Summary of Grant Types Awarded
**Supplemental Figure 1**	Chairperson Prioritization of Research Areas
**Supplemental Table 2**	Summary of Department Resources, Incentives, Benchmarks, and Resident Workforce
**Supplemental Table 3**	Faculty Respondent Characteristics
**Supplemental Figure 2**	Surgeon Specialties
**Supplemental Figure 3**	Relationship Between Contractually Protected Research Time and Outcomes of Interest
**Supplemental Table 4**	Secondary Analyses Examining Relationship Between Individual Factors and Research Productivity

Funding Surgeon-Scientist Research: Department Chair Survey

Thank you for participating in our survey. This data will be used to help us understand factors that contribute to Surgeon Scientist/Clinician Investigator research productivity. Data will be shared publicly in a de-identified and aggregate manner.

This survey has been reviewed by the Vanderbilt University Medical Center (VUMC) Institutional Review Board (IRB) and was approved on 2/10/22 (IRB #220162).

This work is supported by a 2018 Burroughs Wellcome Fund Physician-Scientist Institutional Award to Vanderbilt University (ID: 1018894): "Supporting Careers in Research for Interventional Physicians and Surgeons"

Questions? Contact Paula Marincola Smith, MD, PhD at paula.m.smith@vumc.org

DEPARTMENTAL RESEARCH PRIORITIES

At which institution do you serve as department Chair?

- ◯ Baylor College of Medicine
- ◯ Case Western Reserve Univ. / Cleveland Clinic Lerner
- ◯ Columbia University Health Sciences
- ◯ Cornell University/ Weill Medical College
- ◯ Duke University
- ◯ Emory University
- ◯ Indiana Univ./ Purdue Univ. - Indianapolis
- ◯ Johns Hopkins University
- ◯ Mayo Clinic - Rochester
- ◯ Mount Sinai/ Icahn School Of Medicine
- ◯ New York University School of Medicine
- ◯ Northwestern University - Chicago
- ◯ Oregon Health & Science University
- ◯ Stanford University
- ◯ University of Alabama - Birmingham
- ◯ University of California - Los Angeles
- ◯ University of California - San Diego
- ◯ University of California - San Francisco
- ◯ University of Colorado - Denver
- ◯ University of Michigan
- ◯ University of Minnesota
- ◯ University of North Carolina - Chapel Hill
- ◯ University of Pennsylvania
- ◯ University of Pittsburgh
- ◯ University of Texas Southwestern - Dallas
- ◯ University of Washington
- ◯ University of Wisconsin - Madison
- ◯ Vanderbilt University
- ◯ Washington University - St. Louis
- ◯ Yale University
- ◯ Other

At which other institution(s) do you serve as department Chair? ______________________________

How many attending surgeons are employed by your department?	◯ < 30<br>◯ 30-39<br>◯ 40-49<br>◯ 50-59<br>◯ 60-69<br>◯ 70-79<br>◯ 80-89<br>◯ 90-99<br>◯ >100
What proportion of surgeons in your department currently participate in research in some capacity (including health services, basic science, translational, diversity/equity/inclusion, etc.)?	◯ 0-19%<br>◯ 20-39%<br>◯ 40-59%<br>◯ 60-79%<br>◯ 80-100%<br>◯ Unsure
Which career tracks does your department offer for faculty surgeons?<br><br>Select all that apply.	☐ Clinician-educator<br>☐ Surgeon-scientist / Clinician-investigator*<br>☐ Other<br>(*Terms used interchangeably throughout the survey)
List other career track(s) offered.	__________________________________

For the following questions: How much of a priority does your department consider the following types of research (consider faculty recruitment/funding, departmental growth)?

Basic science research	◯ Not a priority<br>◯ Low priority<br>◯ Medium priority<br>◯ High priority<br>◯ Essential priority
Clinical outcomes research	◯ Not a priority<br>◯ Low priority<br>◯ Medium priority<br>◯ High priority<br>◯ Essential priority
Clinical trials research	◯ Not a priority<br>◯ Low priority<br>◯ Medium priority<br>◯ High priority<br>◯ Essential priority
Diversity/equity/inclusion research	◯ Not a priority<br>◯ Low priority<br>◯ Medium priority<br>◯ High priority<br>◯ Essential priority
Health equity research	◯ Not a priority<br>◯ Low priority<br>◯ Medium priority<br>◯ High priority<br>◯ Essential priority

Health economics research	◯ Not a priority<br>◯ Low priority<br>◯ Medium priority<br>◯ High priority<br>◯ Essential priority
International surgery/global health research	◯ Not a priority<br>◯ Low priority<br>◯ Medium priority<br>◯ High priority<br>◯ Essential priority
Public health research	◯ Not a priority<br>◯ Low priority<br>◯ Medium priority<br>◯ High priority<br>◯ Essential priority
Quality improvement research	◯ Not a priority<br>◯ Low priority<br>◯ Medium priority<br>◯ High priority<br>◯ Essential priority
Translational science research	◯ Not a priority<br>◯ Low priority<br>◯ Medium priority<br>◯ High priority<br>◯ Essential priority
In 2020-2021, about how much National Institute of Health (NIH) research funding did your department receive?	◯ < $3.0 million<br>◯ $3.0-4.9 million<br>◯ $5.0-9.9 million<br>◯ $10.0-14.9 million<br>◯ $15.0-19.9 million<br>◯ $20.0-24.9 million<br>◯ $25.0-29.9 million<br>◯ >$30.0 million
Is your institution a National Cancer Institute (NCI) designated cancer center?	◯ Yes<br>◯ No
What is the level designation of the trauma center at your institution?	◯ Level 1<br>◯ Level 2<br>◯ Level 3<br>◯ None

RESEARCH RESOURCES & SUPPORT

Does your department and/or institution provide the following resources to surgeon-scientist faculty?

Formalized research mentorship for junior faculty
◯ Yes
◯ No

Grant management services
◯ Yes
◯ No

Clinical trial support services to help oversee trial planning and management
◯ Yes
◯ No

Direct early-career funding for junior faculty researchers
◯ Yes
◯ No

On average, how many years of direct financial support does your department or institution guarantee junior faculty before they are expected to receive independent research funding?
◯ < 1
◯ 1 to < 2
◯ 2 to < 3
◯ 3 or more

Grant writing workshops or “mock study sections”
◯ Yes
◯ No

Biostatistics core service (or biostatistics support)
◯ Yes
◯ No

Does your department and/or institution provide incentives/compensation to surgeon-scientist faculty for any of the following?

Grants awarded
◯ Yes
◯ No

How are grants incentivized?
____

Manuscripts published
◯ Yes
◯ No

How are manuscripts incentivized?
____

National presentations
◯ Yes
◯ No

How are national presentations incentivized?
____

Does your department and/or institution have any of the following benchmarks/expectations for surgeon-scientist faculty?

Grants awarded
◯ Yes
◯ No

What are the expectations for grants awarded?
____

Manuscripts published	◯ Yes<br>◯ No
What are the expectations for manuscripts? Any requirement for being first/senior author?	__________________________________
National presentations	◯ Yes<br>◯ No
What are the expectations for presentations?	__________________________________

RESEARCH INVOLVEMENT: SURGERY RESIDENTS

How many categorical general surgery residents are employed at your institution?	◯ < 20<br>◯ 20-29<br>◯ 30-39<br>◯ 40-49<br>◯ 50-59<br>◯ 60-69<br>◯ 70-79<br>◯ 80-89<br>◯ >90
Does your institution/department require categorical general surgery residents to do dedicated research time?	◯ Yes<br>◯ No
On average, how many years do categorical general surgery residents spend dedicated to research?	◯ 0<br>◯ < 1<br>◯ 1 to < 2<br>◯ 2 to < 3<br>◯ 3 or more
Does your department or institution guarantee funding for categorical general surgery residents during their dedicated research time?	◯ Yes<br>◯ No
By what mechanisms does your department or institution fund dedicated research time for residents?<br><br>Select all that apply.	☐ NIH T32 Grant(s)<br>☐ Other Federal Grant(s)<br>☐ Foundation Grant(s)<br>☐ Endowed Scholarship(s)<br>☐ General Departmental Funds<br>☐ Other
Please list other funding mechanism(s).	__________________________________
Thank you for completing Phase 1 (Chair survey) of the Academic Surgeon Survey!<br><br>Please enter the email address of a delegate (e.g. Vice Chair, Administrator) we can work with to distribute the Phase 2 survey to your faculty. You may enter multiple emails.<br><br>Preview the Phase 2 survey at: https://redcap.link/AcademicSurgeons & email any questions to paula.m.smith@vumc.org	__________________________________

Funding Surgeon-Scientist Research: Surgical Faculty Survey

Thank you for participating in our survey. This data will be used to help us understand factors that contribute to Surgeon Scientist/Clinician Investigator research productivity. Data will be shared publicly in a de-identified and aggregate manner.

All questions are optional but encouraged.

Immediately after clicking submit on the final page, you will be taken to an optional screen to enter your information to receive an e-gift card for your participation. Your information will not be linked to your survey response and will not be used for any purpose other than gift card distribution.

If you are not interested in receiving the e-gift card, simply click submit and then close the page.

This survey has been reviewed by the Vanderbilt University Medical Center (VUMC) Institutional Review Board (IRB) and was approved on 2/10/22 (IRB #220162).

This work is supported by a 2018 Burroughs Wellcome Fund Physician-Scientist Institutional Award to Vanderbilt University (ID: 1018894): "Supporting Careers in Research for Interventional Physicians and Surgeons"

Questions? Contact Paula Marincola Smith, MD, PhD at paula.m.smith@vumc.org

---

At which institution are you currently employed?

- ◯ Baylor College of Medicine
- ◯ Case Western Reserve Univ. / Cleveland Clinic Lerner
- ◯ Columbia University Health Sciences
- ◯ Cornell University/ Weill Medical College
- ◯ Duke University
- ◯ Emory University
- ◯ Indiana Univ./ Purdue Univ. - Indianapolis
- ◯ Johns Hopkins University
- ◯ Mayo Clinic - Rochester
- ◯ Mount Sinai/ Icahn School Of Medicine
- ◯ New York University School of Medicine
- ◯ Northwestern University - Chicago
- ◯ Oregon Health & Science University
- ◯ Stanford University
- ◯ University of Alabama - Birmingham
- ◯ University of California - Los Angeles
- ◯ University of California - San Diego
- ◯ University of California - San Francisco
- ◯ University of Colorado - Denver
- ◯ University of Michigan
- ◯ University of Minnesota
- ◯ University of North Carolina - Chapel Hill
- ◯ University of Pennsylvania
- ◯ University of Pittsburgh
- ◯ University of Texas Southwestern - Dallas
- ◯ University of Washington
- ◯ University of Wisconsin - Madison
- ◯ Vanderbilt University
- ◯ Washington University - St. Louis
- ◯ Yale University
- ◯ Other

At which other institution(s) are you employed?

____

What is your current surgical specialty?<br>Select all that apply	☐ Abdominal Transplant Surgery<br>☐ Acute Care Surgery<br>☐ Adult Cardiac Surgery<br>☐ Bariatric Surgery<br>☐ Burn Surgery<br>☐ Colon & Rectal Surgery<br>☐ Nose, and Throat (ENT) Surgery<br>☐ Endocrine Surgery<br>☐ General Surgery<br>☐ Hand Surgery<br>☐ Minimally Invasive Surgery<br>☐ Neurologic Surgery<br>☐ Oral/Maxillofacial Surgery<br>☐ Orthopedic Surgery<br>☐ Pediatric Cardiac Surgery<br>☐ Pediatric General Surgery<br>☐ Plastic/Reconstructive Surgery<br>☐ Surgical Critical Care<br>☐ Surgical Oncology: Breast/Soft Tissue<br>☐ Surgical Oncology: Complex Gastrointestinal<br>☐ Thoracic Surgery<br>☐ Trauma Surgery<br>☐ Urologic Surgery<br>☐ Vascular Surgery
How many years are you into your career/how many years ago did you finish training (residency/fellowship)?	________________________
What is your current faculty position?	◯ Instructor<br>◯ Assistant Professor<br>◯ Associate Professor<br>◯ Professor<br>◯ Other
Please list other faculty position(s).	________________________
How would you classify your faculty position?	◯ Research track<br>◯ Clinician-educator track<br>◯ Other
Please list other faculty position track(s).	________________________
How would you best describe your current research?<br><br>Select all that apply	☐ Basic Science<br>☐ Clinical Trials<br>☐ Clinical Outcomes<br>☐ Diversity/Equity/Inclusion<br>☐ Health Economics<br>☐ Health Equity<br>☐ International Surgery/Global Health<br>☐ Public Health<br>☐ Quality Improvement<br>☐ Social Sciences<br>☐ Translational Science<br>☐ Other<br>☐ Not currently involved with research
Please list other research fields.	________________________

During which career stage did you begin to identify as a surgeon scientist/clinical investigator?

- ◯ Undergraduate or earlier
- ◯ Medical/graduate school
- ◯ Residency
- ◯ Fellowship
- ◯ Faculty
- ◯ Other

Please describe. __________________________________

What graduate degree(s) do you have?

Select all that apply

- ☐ MD
- ☐ DO
- ☐ PhD
- ☐ MS
- ☐ MA
- ☐ MSCI
- ☐ MPH
- ☐ MBA
- ☐ Other

Please list other degree(s). __________________________________

With what gender do you identify?

Select all that apply

- ☐ Male
- ☐ Female
- ☐ Non-binary
- ☐ Other
- ☐ Prefer not to say

Please list other gender identities. __________________________________

With what race(s) do you identify?

Select all that apply

- ☐ White
- ☐ Black/African American
- ☐ Asian
- ☐ Native Hawaiian/Pacific Islander
- ☐ American Indian/Alaskan Native
- ☐ Other
- ☐ Prefer not to say

Please list other race identities. __________________________________

With what ethnicity do you identify?

Select all that apply

- ☐ Hispanic/Latino
- ☐ Not Hispanic/Latino
- ☐ Prefer not to say

Do you have children or dependents?

- ☐ Yes
- ☐ No
- ☐ Prefer not to say

How much student debt do you/did you have upon graduation from training (residency or fellowship)?

- ◯ < $10,000
- ◯ $10,000.01 - 50,000
- ◯ $50,000.01 - 100,000
- ◯ $100,000.01 - 150,000
- ◯ $150,000.01 - 200,000
- ◯ $200,000.01 - 250,000
- ◯ $250,000.01 - 300,000
- ◯ $300,000.01 - 350,000
- ◯ $350,000.01 - 400,000
- ◯ >$400,000
- ◯ Prefer not to say

If you did have student loan debt upon graduation from training, did you/are you participating in any of the following loan repayment programs?

- ☐ Public Service Loan Forgiveness Program
- ☐ VA Student Loan Repayment Program
- ☐ NIH Loan Repayment Program
- ☐ Private sponsor program
- ☐ Other

Select all that apply

Please list other loan repayment program(s).

____

RESIDENCY

How many years of dedicated research time did you do during residency?

- ◯ 0
- ◯ < 1
- ◯ 1 to < 2
- ◯ 2 to < 3
- ◯ 3 to < 4
- ◯ 4+

How would you best describe your research during residency?

Select all that apply

- ☐ Basic Science
- ☐ Clinical Trials
- ☐ Clinical Outcomes
- ☐ Diversity/Equity/Inclusion
- ☐ Health Economics
- ☐ Health Equity
- ☐ International Surgery/Global Health
- ☐ Public Health
- ☐ Quality Improvement
- ☐ Social Sciences
- ☐ Translational Science
- ☐ Other

Please describe other research fields.

____

Did you take formal coursework (either degree-seeking or non-degree-seeking) on the topic of grant writing during your dedicated research time during residency?

- ◯ Yes
- ◯ No

How many research grants did you apply for during your dedicated research time during residency (including NIH/federal, foundation/non-federal, and internal/institutional awards)?

____

Did you have formal assistance preparing your grant application(s) in the form of mock study sections, grant writing workshops, etc. (not including informal feedback such as from your own research mentor or colleagues)?

- ◯ Yes
- ◯ No

How many grant applications were successfully funded?

____

Please select the grant types which were applied for and funded.
Select all that apply.

- ☐ NIH NRSA F32
- ☐ Other federal award [NIH/VA/DoD/etc.]
- ☐ Foundation award [AAS/ACS/etc.]
- ☐ Internal/Institutional award
- ☐ Other

How was your research time funded?

Select all that apply.

- ☐ NIH NRSA T32
- ☐ Institution/Department
- ☐ Service obligations
- ☐ Other

How many peer-reviewed papers did you publish as a result of the research you conducted during residency?

____

For how many of these papers were you first or senior author?

____

FELLOWSHIP

Did you complete fellowship training?	◯ Yes<br>◯ No
How many months of dedicated research time did you do during fellowship?	◯ 0<br>◯ 1 to < 3<br>◯ 3 to < 6<br>◯ 6 to < 9<br>◯ 9 to < 12<br>◯ 12+
How would you best describe your research during fellowship?<br><br>Select all that apply	☐ Basic Science<br>☐ Clinical Trials<br>☐ Clinical Outcomes<br>☐ Diversity/Equity/Inclusion<br>☐ Health Economics<br>☐ Health Equity<br>☐ International Surgery/Global Health<br>☐ Public Health<br>☐ Quality Improvement<br>☐ Social Sciences<br>☐ Translational Science<br>☐ Other
Please describe other research fields.	__________________________________
Did you take formal coursework (either degree-seeking or non-degree-seeking) on the topic of grant writing during your dedicated research time during fellowship?	◯ Yes<br>◯ No
How many research grants did you apply for during your dedicated research time during fellowship (including NIH/federal, foundation/non-federal, and internal/institutional awards)?	__________________________________
Did you have formal assistance preparing your grant application(s) in the form of mock study sections, grant writing workshops, etc. (not including informal feedback such as from your own research mentor or colleagues)?	◯ Yes<br>◯ No
How many grant applications were successfully funded?	__________________________________
Please select the grant types which were applied for and funded.<br>Select all that apply.	☐ NIH NRSA F32<br>☐ Other federal award [NIH/VA/DoD/etc.]<br>☐ Foundation award [AAS/ACS/etc.]<br>☐ Internal/Institutional award<br>☐ Other
How was your research time funded?<br><br>Select all that apply.	☐ NIH NRSA T32<br>☐ Institution/Department<br>☐ Service obligations<br>☐ Other

How many peer-reviewed papers did you publish as a result of the research you conducted during fellowship? __________________________________

For how many of these papers were you first or senior author? __________________________________

FACULTY

As a faculty member, how many research grants have you applied for (including NIH/federal, foundation/non-federal, and internal/institutional awards)?

____

Did you take formal coursework (either degree-seeking or non-degree-seeking) on the topic of grant writing as a faculty member?

◯ Yes
◯ No

How many grant applications were successfully funded?

____

Please select the types of grants which were applied for and funded.
Select all that apply.

☐ NIH K- series award
☐ NIH R- series award (R01/R21/etc.)
☐ NIH P- or U- series award
☐ VA Early Career Development Award
☐ VA Merit Review
☐ Other federal award [DoD, other NIH/VA mechanisms/etc.]
☐ Foundation award [AAS/ACS/etc.]
☐ Internal/Institutional award
☐ Other

During how many years of your faculty career have you had independent research funding?

____

How many peer-reviewed papers have you published as a result of the research you conducted as a faculty member?

____

For how many of these papers were you first or senior author?

____

According to your contract, what percentage of your time is "protected" for the following activities?

Answer in a whole integer (i.e., fifteen percent = "15")

Research

____
(Round to the nearest whole integer)

Administrative tasks

____
(Round to the nearest whole integer)

Patient care

____
(Round to the nearest whole integer)

Teaching

____
(Round to the nearest whole integer)

In reality, what percentage of your time would you estimate is dedicated to the following activities?

Answer in a whole integer (i.e., fifteen percent = "15")

Research	__________________________________ (Round to the nearest whole integer)
Administrative tasks	__________________________________ (Round to the nearest whole integer)
Patient care	__________________________________ (Round to the nearest whole integer)
Teaching	__________________________________ (Round to the nearest whole integer)
How many hours per week do you work, on average?	__________________________________
How often do you take call "in house"? (# calls per month)	__________________________________
How often do you take call from home? (# calls per month)	__________________________________
Do you have a formal research mentor, assigned to you by your department or institution?	◯ Yes<br>◯ No
Do you have an informal research mentor, which you identified independently?	◯ Yes<br>◯ No
How much less or more supportive do you feel your department/institution is of early- and mid-career surgeon scientists, compared to peer institutions?	◯ Much less supportive<br>◯ Less supportive<br>◯ Equally supportive<br>◯ More supportive<br>◯ Much more supportive
How supported do you feel by your colleagues/partners in your research endeavors?	◯ Very unsupported<br>◯ Unsupported<br>◯ Neither supported nor unsupported<br>◯ Supported<br>◯ Very supported

Final Thoughts

How satisfied are you that you have been able to attain your overall research goals as a surgeon-scientist?

◯ Very unsatisfied
◯ Unsatisfied
◯ Neither satisfied nor unsatisfied
◯ Satisfied
◯ Very satisfied

What has been the greatest help to your success/productivity as a surgeon scientist?

Select up to three.

☐ Biostatistics Support
☐ Grant writing support services
☐ Mentorship (formal or informal)
☐ Collaboration
☐ Core services
☐ Direct early career funding from department/institution
☐ Personal/family support
☐ Other (please describe)

What other types of research support have contributed to your success/productivity as a surgeon-scientist?

____

What has been the greatest barrier to your success/productivity as a surgeon scientist?

Select up to three.

☐ Clinical duties/patient care
☐ Administrative work
☐ Teaching obligations
☐ Lack of biostatistics support
☐ Lack of grant writing support services
☐ Lack of mentorship (formal or informal)
☐ Lack of collaboration
☐ Lack of core services
☐ Lack of direct early career funding from department/institution
☐ Personal/family considerations
☐ Other (please describe)

What other barriers have hindered your success/productivity as a surgeon-scientist?

____

Additional thoughts on promoting surgeon-scientist success?

____

Thank you for your invaluable contributions to this survey. Immediately after clicking submit, you will be taken to an optional screen to enter your information to receive an e-gift card for your participation. Your information will not be linked to your survey response and will not be used for any purpose other than gift card distribution.

If you are not interested in receiving the e-gift card, simply click submit below and then close the page.

Please click submit to ensure your response is recorded.

Number of Grants Awarded	
**Award Type**	**Number (%)**
**NIH K-Series**	65 (14.6)
**NIH R-Series**	77 (17.3)
**NIH P- or U- Series**	19 (4.3)
**VA Early Career Development**	12 (2.7)
**VA Merit**	20 (4.5)
**Other Federal**	57 (12.8)
**Foundation**	144 (32.4)
**Internal/Institutional**	143 (32.2)
**Other**	39 (8.8)

Supplemental Table 1: Summary of Grant Types Awarded. Abbreviations: NIH = National Institutes of Health, VA = Veterans Affairs.

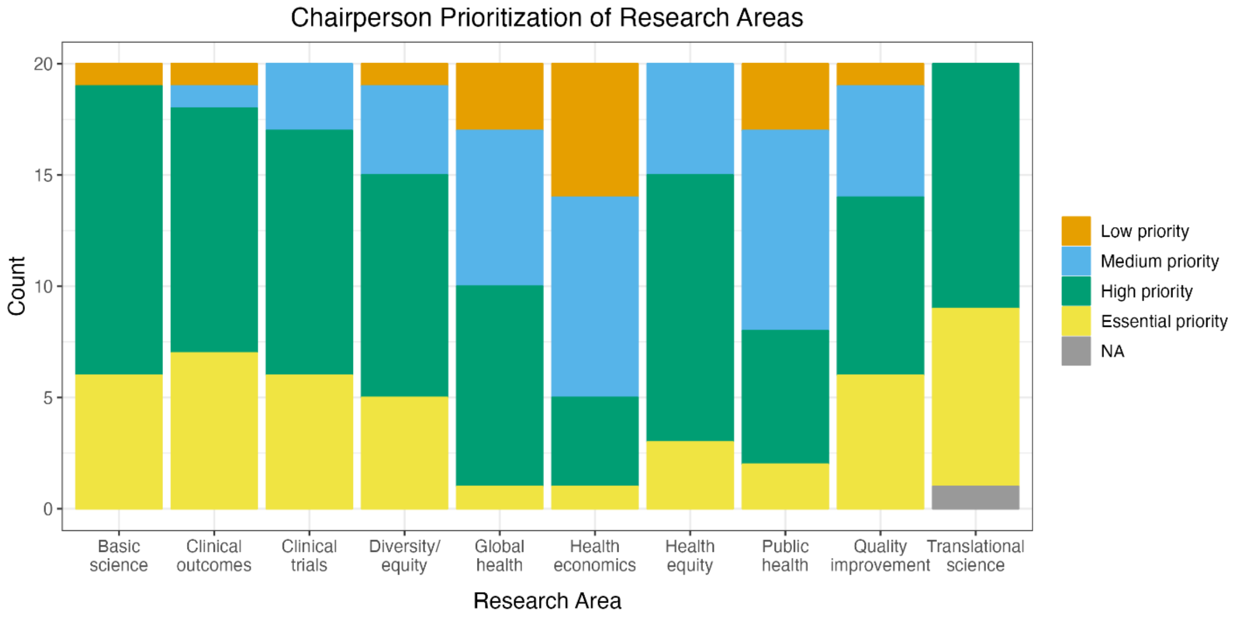

Supplemental Figure 1: Chairperson Prioritization of Research Areas.

	Number of Respondents (N=20)	
	Count (n)	%
**Percent of Surgical Faculty Involved in Research**		
0-19%	1	5.0
20-39%	4	20.0
40-59%	4	20.0
60-79%	3	15.0
80-100%	8	40.0
**Research Support Services Offered**		
**Formalized Research Mentorship Offered**		
No	1	5.0
Yes	17	85.0
Missing	2	10.0
**Grant Management Services Offered**		
No	0	0.0
Yes	19	95.0
Missing	1	5.0
**Clinical Trial Support Services Offered**		
No	2	10.0
Yes	17	85.0
Missing	1	5.0
**Direct Early Career Funding Offered**		
No	0	0.0
Yes	19	95.0
Missing	1	5.0
**Years of Direct Financial Support Offered**		
<1	0	0.0
1 to <2	0	0.0
2 to <3	9	45.0
3 or more	10	50.0
Missing	1	5.0
**Grant Writing Workshops Offered**		
No	1	5.0
Yes	18	90.0
Missing	1	5.0
**Biostatistics Support Offered**		
No	0	0.0
Yes	18	90.0
Missing	2	10.0
**Incentives Offered**		
**Incentives Offered for Grants Awarded**		
No	7	35.0
Yes	12	60.0
Missing	1	5.0
**Incentives Offered for Manuscripts Published**		
No	14	70.0

Yes	5	25.0
Missing	1	5.0
Incentives Offered for National Presentations		
No	16	80.0
Yes	3	15.0
Missing	1	5.0
Benchmarks Provided		
Benchmarks for Grants Awarded		
No	9	45.0
Yes	10	50.0
Missing	1	5.0
Benchmarks for Manuscripts Published		
No	12	60.0
Yes	7	35.0
Missing	1	5.0
Benchmarks for National Presentations		
No	15	75.0
Yes	4	20.0
Missing	1	5.0
Residents in Research Workforce		
Number of General Surgery Residents		
<40	0	0.0
40-49	3	15.0
50-59	10	50.0
60-69	0	0.0
70-79	3	15.0
80-89	0	0.0
≥90	3	15.0
Missing	1	5.0
Required Research Time ≥ 1 Year for Categorical General Surgery Residents		
No	9	45.0
Yes	10	50.0
Missing	1	5.0
Years Residents Spend in Research		
0	0	0.0
<1	1	5.0
1 to <2	3	15.0
2 to <3	14	70.0
3 or more	1	5.0
Missing	1	5.0
Guaranteed Funding for Residents in Research		
No	1	5.0
Yes	18	90.0
Missing	1	5.0
Mechanisms of Funding Residents in Research		
General Department Funds	16	80.0
NIH T32 grants	14	70.0

Other Federal Grants	13	65.0
Foundation Grants	13	65.0
Endowed Scholarships	12	60.0
Other	4	20.0

Supplemental Table 2: Summary of Department Resources, Incentives, Benchmarks, and Resident Workforce. Abbreviations: NIH = National Institutes of Health.

	Number of Respondents (N=444)	
	Count (n)	%
Institution		
Vanderbilt University	86	19.4
University of Michigan	55	12.4
University of Wisconsin – Madison	48	10.8
University of Pennsylvania	44	9.9
University of Texas Southwestern – Dallas	31	7.0
Duke University	30	6.8
Indiana University/Purdue University – Indianapolis	24	5.4
Baylor College of Medicine	23	5.2
University of Alabama – Birmingham	19	4.3
Yale University	18	4.1
Stanford University	15	3.4
Northwestern University – Chicago	14	3.2
University of California – Los Angeles	12	2.7
Missing	25	5.6
Gender		
Female	143	32.2
Male	281	63.3
Non-Binary	2	0.5
Prefer not to say	18	4.1
Race		
Asian	70	15.8
Black/African American	11	2.5
Other	11	2.5
White	325	73.2
Prefer not to say	26	5.9
Missing	1	0.2
Ethnicity		
Hispanic/Latino	17	3.8
Not Hispanic/Latino	394	88.7
Prefer not to say	33	4.1
Children/Dependents		
No	73	16.4
Yes	353	79.5
Prefer not to say	18	4.1
Years Since Finishing Training		
0-5 years	113	25.5
5-10 years	77	17.3
10-15 years	68	15.3
15-20 years	54	12.2
20-25 years	43	9.7
25-30 years	27	6.1
30-35 years	17	3.8
35-40 years	13	2.9

40-45 years	5	1.1
45-50 years	4	0.9
Missing	23	5.2
Median [Q1-Q3]	10.0 [4.0-20.0]	n=422
Current Faculty Position		
Instructor	8	1.8
Assistant Professor	168	37.8
Associate Professor	117	26.4
Professor	142	32.0
Other	8	1.8
Missing	1	0.2
Faculty Career Track		
Clinician-Educator Track	300	67.6
Research Track	107	24.1
Other	35	7.9
Missing	2	0.5
Clinical Degree		
MD	420	94.6
DO	6	1.4
Other (e.g. MBBS, DMD, DDS)	18	4.1
Additional Degrees		
PhD	38	8.6
MS	77	17.3
MA	3	0.7
MSCI	8	1.8
MPH	44	9.9
MBA	15	3.4
Other	27	6.1
Hours Worked		n
Hours worked per week, median [Q1-Q3]	60.0 [57.5-75.0]	395
Began to Identify as a Surgeon-Scientist		
Undergraduate or Earlier	43	9.7
Medical/Graduate School	96	21.6
Residency	168	37.8
Fellowship	44	9.9
Faculty	80	18.0
Other	13	2.9
Debt Upon Graduation from Training		
≤$10,000	125	28.2
$10,000.01 – 50,000.00	55	12.4
$50,000.01 – 100,000.00	44	9.9
$100,000.01 – 150,000.00	46	10.4
$150,000.01 – 200,000.00	42	9.5
$200,000.01 – 250,000.00	37	8.3
$250,000.01 – 300,000.00	29	6.5
$300,000.01 – 350,000.00	13	2.9
$350,000.01 – 400,000.00	11	2.5

>400,000.00	20	4.5
Prefer not to say	21	4.7
Missing	1	0.2
Participation in Loan Repayment Program		
NIH Loan Repayment Program	32	7.2
Private Sponsor	13	2.9
Public Service Loan Forgiveness	82	18.5
VA Student Loan Repayment Program	2	0.5
Other	21	4.7

Supplemental Table 3: Faculty Respondent Characteristics. Abbreviations: MD = Medical Doctor, DO = Doctor of Osteopathic Medicine, MBBS = Bachelor of Medicine and Bachelor of Surgery, DMD = Doctor of Dental Medicine, DDS = Doctor of Dental Surgery, PhD = Doctor of Philosophy, MS = Masters of Science, MA = Master of Arts, MSCI = Master of Science in Clinical Investigation, MPH = Master of Public Health, MBA = Master of Business Administration, NIH = National Institutes of Health, VA = Veterans Affairs

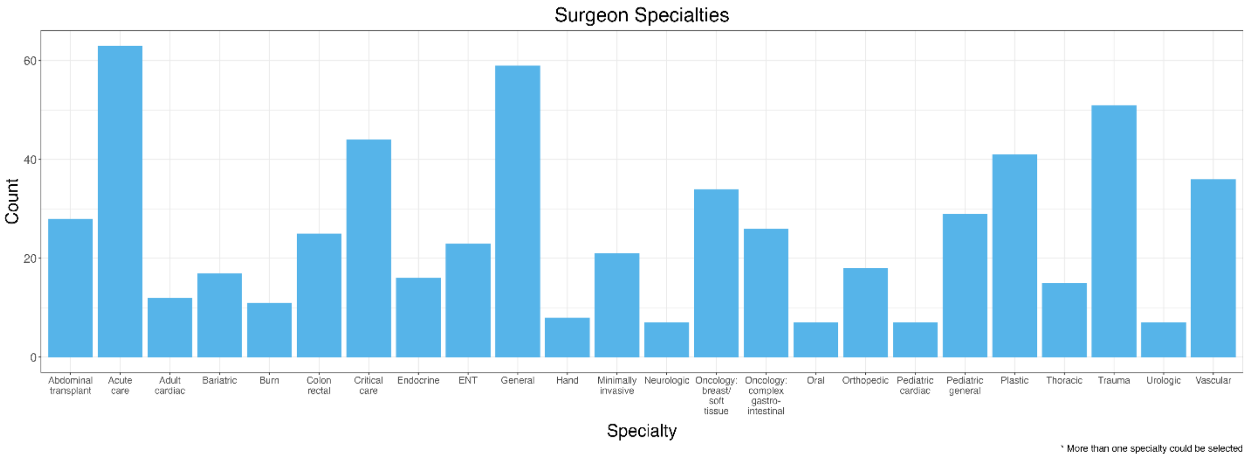

Supplemental Figure 2: Surgeon Specialties.

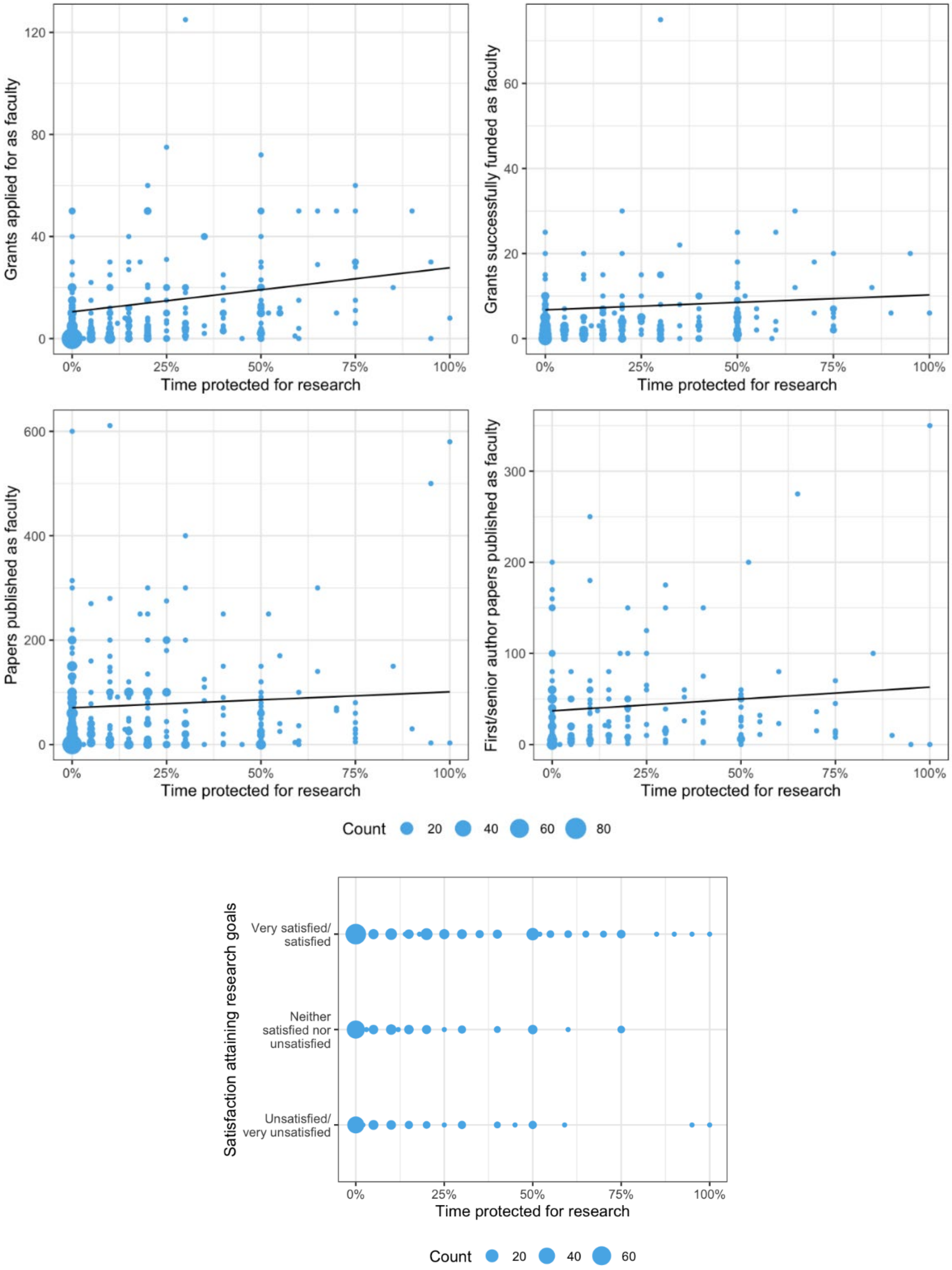

Supplemental Figure 3: Relationship Between Contractually Protected Research Time and Outcomes of Interest.

	Grants Applied for as Faculty, IRR	Grants Funded as Faculty, IRR	Papers Published as Faculty, IRR	First/Senior Author Papers Published as Faculty, IRR	Satisfaction in Research as Faculty, OR
Demographic Factors					
Gender, Male	1.35 (0.95-1.91)	1.02 (0.75-1.38)	1.19 (0.88-1.62)	1.06 (0.77-1.46)	1.33 (0.87-2.04)
Gender, Non-Binary	0.87 (0.11-6.86)	0 (0-Inf)	3.24 (0.53-19.71)	0.54 (0.07-4.25)	2.06 (0.17-25.51)
Race, Black/African American	0.49 (0.17-1.43)	0.65 (0.23-1.84)	0.48 (0.19-1.17)	0.68 (0.20-2.31)	0.48 (0.16-1.47)
Race, Asian	1.30 (0.85-1.98)	1.06 (0.75-1.50)	1.02 (0.70-1.50)	1.02 (0.69-1.52)	1.42 (0.82-2.45)
Race, Other	1.00 (0.35-2.87)	0.99 (0.35-2.74)	0.56 (0.24-1.32)	0.38 (0.13-1.08)	0.27 (0.08-0.87)
Ethnicity, Hispanic/Latino	0.74 (0.35-1.56)	0.51 (0.26-1.01)	0.98 (0.48-2.00)	1.19 (0.54-2.66)	1.41 (0.50-4.00)
Children/Dependents, Yes	1.77 (1.16-2.72)	1.15 (0.77-1.73)	1.22 (0.83-1.79)	1.11 (0.73-1.70)	1.05 (0.62-1.77)
Training Factors					
Research Time During Residency	2.20 (1.49-3.26)	1.45 (1.01-2.08)	1.47 (1.06-2.05)	1.52 (1.08-2.15)	1.54 (0.98-2.43)
Grant Writing Course During Training	1.53 (1.05-2.24)	1.41 (1.02-1.94)	1.60 (1.12-2.29)	1.70 (1.19-2.43)	1.56 (0.92-2.65)
Assistance Preparing Grants in Training	0.88 (0.55-1.41)	0.84 (0.55-1.28)	1.19 (0.75-1.90)	1.17 (0.71-1.92)	1.28 (0.64-2.57)
Extramural Funding During Training	2.22 (1.55-3.20)	1.74 (1.32-2.28)	1.33 (0.98-1.82)	1.24 (0.89-1.72)	1.89 (1.19-3.01)
Faculty-Specific Factors					
Research Track	4.24 (3.02-5.95)	2.90 (2.22-3.80)	2.01 (1.47-2.75)	1.74 (1.24-2.44)	3.17 (1.94-5.18)
Shift Work	0.88 (0.61-1.28)	0.94 (0.69-1.29)	1.08 (0.78-1.49)	0.99 (0.69-1.41)	0.92 (0.58-1.47)
<$100k debt, no repayment program	0.75 (0.40-1.42)	0.77 (0.46-1.29)	0.96 (0.54-1.69)	0.87 (0.48-1.59)	0.50 (0.20-1.21)

>$100k debt, repayment program	0.64 (0.32-1.26)	0.71 (0.40-1.25)	0.65 (0.36-1.19)	0.64 (0.33-1.21)	0.39 (0.15-0.98)
>$100k debt, no repayment program	0.50 (0.26-0.99)	0.55 (0.31-0.96)	0.85 (0.46-1.55)	0.77 (0.41-1.45)	0.34 (0.14-0.87)
Grant Writing Course as Faculty	1.77 (1.20-2.59)	1.10 (0.82-1.48)	1.22 (0.87-1.71)	1.16 (0.83-1.63)	1.71 (1.03-2.82)
Average Hours Worked per Week as Faculty	1.02 (1.00-1.03)	1.01 (1.00-1.02)	1.01 (1.00-1.02)	1.01 (1.00-1.02)	1.00 (0.98-1.01)
Informal Research Mentor, Yes	2.27 (1.65-3.11)	2.03 (1.54-2.69)	1.50 (1.13-2.00)	1.24 (0.90-1.70)	2.76 (1.79-4.26)
Perception of Departmental/Institutional Support					
Less Supportive	0.57 (0.25-1.31)	0.54 (0.25-1.15)	1.31 (0.64-2.71)	0.73 (0.34-1.54)	1.21 (0.44-3.36)
Equally Supportive	0.52 (0.24-1.13)	0.59 (0.28-1.26)	1.12 (0.57-2.22)	0.81 (0.39-1.70)	1.47 (0.56-3.83)
More Supportive	0.99 (0.44-2.20)	0.80 (0.38-1.69)	1.64 (0.82-3.29)	0.79 (0.38-1.68)	2.89 (1.08-7.71)
Much More Supportive	1.16 (0.50-2.70_	0.80 (0.37-1.73)	2.33 (1.14-4.76)	1.32 (0.61-2.87)	6.44 (2.25-18.45)
Perception of Colleague Support					
Unsupported	1.27 (0.55-2.93)	1.21 (0.55-2.67)	1.14 (0.53-2.45)	1.39 (0.60-3.24)	0.65 (0.20-2.07)
Neither Supported nor Unsupported	1.55 (0.78-3.11)	1.43 (0.74-2.76)	1.11 (0.59-2.10)	1.05 (0.51-2.15)	1.61 (0.62-4.19_
Supported	1.55 (0.81-2.98)	1.52 (0.81-2.83)	1.31 (0.71-2.41)	1.41 (0.73-2.73)	4.26 (1.67-10.84)
Very Supported	3.46 (1.73-6.94)	2.63 (1.37-5.05)	2.11 (1.11-3.99)	2.01 (1.00-4.04)	13.71 (4.93-38.14)

Supplemental Table 4: Secondary Analyses Examining Relationship Between Individual Factors and Research Productivity. Abbreviations: IRR = Incidence Rate Ratio, OR = Odds Ratio.
